# Supplementary material for: Microbiomes in the Challenger Deep slope and bottom-axis sediments
Source: Nat Commun. 2022 Mar 21;13:1515. doi: 10.1038/s41467-022-29144-4 (PMC8938466; doi:10.1038/s41467-022-29144-4)
Supplement: Supplementary file 1 — Supplementary Information [file 41467_2022_29144_MOESM1_ESM.pdf]

Supplementary Information: Microbiomes in the Challenger Deep slope and bottom-axis sediments

Author List:

Ying-Li Zhou[1,2#], Paraskevi Mara[3#], Guo-Jie Cui[1,2], Virginia P. Edgcomb[3], Yong Wang[1,4*]

Affiliations:

[1]Institute of Deep-Sea Science and Engineering, Chinese Academy of Sciences, Sanya, Hainan, China

[2]University of the Chinese Academy of Sciences, Beijing, China

[3]Department of Geology and Geophysics, Woods Hole Oceanographic Institution, Woods Hole, MA, USA

[4]Institute for Ocean Engineering, Shenzhen International Graduate School, Tsinghua University, Shenzhen, China

# These authors contributed equally

***Correspondence to**: Yong Wang, wangy@idsse.ac.cn

Supplementary Discussion

Oxygen, ammonium, TOC and TN concentrations

We suggest that the O_2 depletion detected at the deeper depths (> 21 cmbsf) of bottom-axis sediments can be from the intense AOA activity and organic matter degradation that occur at the surface of bottom-axis sediments (this study and [1, 2]). The higher cell density reported in the surface bottom-axis sediments relative to slope sites[2] can contribute to O_2 depletion due to the fast consumption of O_2 as preferable electron acceptor for aerobic respiration.

Sediments collected from slope sites had similar NH_4^+ concentrations (up to 2.5 μM) throughout the examined sediments horizons, and were 5-fold lower compared to those from bottom-axis sediments (Supplementary Fig. 1b, Supplementary Data 1). The detected concentrations of NH_4^+ and NO_3^- in porewaters suggest a nitrate-ammonium transition zone in the bottom-axis sediments, with NH_4^+ concentrations increasing and NO_3^- levels decreasing sharply at depths below 10 cmbsf.

The total organic carbon (TOC)/total nitrogen (TN) molar ratios in CD sediments ranged from 3 to ~ 9.4 (Supplementary Data 1). Our TOC/TN ratios are consistent with previous studies from the Challenger Deep[3] and indicate marine algae as potential source of organic matter at the sediment surface.

Description of the dominant CD microbial phyla

Most assigned CD MAGs were affiliated with known prokaryotic phyla; however, 21 MAGs were sorted to the recently described phyla KSB1, UBP7, UBP7_A, Binatota and GCA-001730085[4, 5] whose ecological role especially in hadal habitats remains unclear. The most widely distributed bacterial and archaeal CD MAGs were B6T1B5 and B7T1B11, assigned to

Gemmatimonadota and Thaumarchaeota, respectively, detected in 32/37 metagenomes (Fig. 2a and Supplementary Data 4). Proteobacteria, Chloroflexota and Planctomycetota were also dominant in our MAGs (Fig. 2a and Supplementary Data 4) as described elsewhere in Mariana Trench[6].

Carbohydrate-Active Enzymes involved in cell wall remodeling and organic matter degradation, and mechanisms of protein recycling in CD

To predict gene functions in the CD metagenomes, we searched the metagenomic reads against the NCBI_nr database and parsed the data using the SEED database[7]. The annotated metagenomic reads were assigned to various functional protein families in the SEED database, with 61% of the reads being affiliated with proteins related to carbohydrate utilization (Supplementary Fig. 12). To further elucidate the potential for carbohydrate utilization in CD sediments, we searched our protein data for Carbohydrate-Active Enzymes (CAZymes) [8].

The largest group of CAZymes encoded by all our MAGs was Glycosyl Transferases (GT) involved in the synthesis of mono- and polysaccharides, glycoconjugates and the remodeling of the microbial cell wall[9, 10] (Supplementary Fig. 10). GT2 and GT4 were the most abundant GT families in CD sediments. The carbohydrate esterase families CE1 and CE4, which include hydrolases and deacetylases responsible for the degradation of peptidoglycan[11], were identified in 52% and 63% our MAGs, respectively (Supplementary Data 6). The abundance of CAZymes differed between the phyla detected in our CD samples (Supplementary Fig. 10c-d). Of note are Patescibacteria that have a small genome but a higher percentage of genes encoding CAZymes (Supplementary Fig. 10d). We also found that polysaccharide lyases (PL; cleave glycosidic bonds)

were encoded in low-abundance phyla (< 1% of relative abundance) such as Latescibacteria and Poribacteria (Supplementary Fig. 11).

Additional known roles of GT gene families are reported for deep-sea piezotolerant bacteria and involve production of biosurfactants for the biodegradation of complex hydrocarbons[12], and chemoenzymatic glycan modifications essential for biofilm formation[13]. However, these additional roles require further investigation in CD sediments. The wide repertoire of CAZymes and peptidases identified in our data indicates that heterotrophic microbes in CD sediments have the potential to utilize simple and complex carbohydrates, and polysaccharides for energy gain.

Evidence for protein recycling was identified in 52 MAGs encoding the bacterial ubiquitin-like protein Pup, as well as other proteins of the Pup-proteasome degradation pathway, which post-translationally modifies and degrades proteins, similarly (albeit genetically unrelated) to ubiquitination[14, 15]. The Pup-proteasome degradation pathway was identified in Actinobacteria, and in > 70% of our MAGs from ammonia-oxidizing archaea (AOA; Thaumarchaeota) and nitrite-oxidizing bacteria (NOB; mainly Nitrospinota and Nitrospirota). Pup-proteasome degradation is closely connected to nitrogen metabolism and contributes to amino acid recycling into nitrogenous precursors especially under nitrogen limiting conditions[16]. Identification of genes encoding the Pup proteasome primarily in NOBs and AOAs suggests a potential active mechanism that can sustain autotrophs that depend on inorganic nitrogen substrates for survival under the challenging nitrogen-related conditions that exist in CD (see Results and Discussion Geochemistry of Challenger Deep (CD) sediments).

Degradation of alkenes and aromatic compounds in CD

Other forms of heterotrophy that sustain sediment microbial communities in deep and remote settings include aerobic and/or anaerobic degradation of aromatic compounds. We detected four dioxygenase genes (*catA, catE, pcaH* and *ligB*)[17] that encode enzymes for the cleavage of aromatic rings (Fig. 3a), with *catE* being the most abundant gene identified in 119/586 of our MAGs. The uncharacterized bacterial phylum Binatota and SAR324 contained most of the identified aromatic degradation genes (Fig. 3a).

The *box* genes (*boxB*, *boxC*, and *boxD*) responsible for the de-aromatization and ring-cleavage steps during benzoate degradation (*box* pathway)[18] were present in 85 MAGs (Supplementary Data 7). The *box* pathway combines steps of aerobic and anaerobic degradation, also known as *hybrid* benzoate degrdation[18]. Although the reported genes in this study are predominantly associated with aerobic and hybrid degradation of aromatic hydrocarbons/benzoate, some MAGs contained marker genes of strictly anaerobic hydrocarbon degradation (e.g. benzoyl-CoA reductase and 4-hydroxybenzoyl-CoA reductase) previously identified in facultative anaerobes[19].

Alkane degrading genes *alkM* and/or *ladA* were identified in 40 MAGs from both slope and bottom-axis sediments and were affiliated with Euryarchaeota, Chloroflexota, Gammaproteobacteria and Myxococcota (Fig. 3a). Previous studies on the CD water column have shown a shift in microbial metabolic potential towards hydrocarbon degradation at water depths > 10,400 m, where n-alkanes are thought to be synthesized in situ and/or released from subsurface sediments to the water column[20]. We suggest that heterotrophic microbial communities in CD sediments contribute in the biological transformation of simple and/or complex hydrocarbons for energy gain, and in turn, provide simple hydrocarbon substrates (e.g. aliphatic n-alkanes) to the

hydrocarbon-degrading microbial populations which are present in the water column at ~11 km water depth in CD[20].

Glycolysis and amino acid metabolism

The annotated metagenomic reads indicated the potential for glycolysis in the CD sediments based on prediction of key functional genes such as glucokinase (*glk*), phosphofructokinase (*pfk*), and pyruvate kinase (*pyk*). Functional genes related to glycolysis were found in ~65% of the CD genomes and were also abundant in all metagenomes and metatranscriptomes (Figs 3a-c; Supplementary Data 7). Evidence of C3 and C4 metabolite interconversions using the phosphoenolpyruvate (PEP)-oxaloacetate-pyruvate node[21], was found in the surficial CD sediments according to the detection of functional genes encoding the phosphoenolpyruvate carboxykinase, pyruvate orthophosphate dikinase and malate dehydrogenase. This could indicate replenishing of tricarboxylic acid cycle (TCA) intermediates near the CD sediment surface where ephemeral oxygen is available.

Genes involved in the metabolism and fermentation of amino acids have been also detected in our CD MAGs. Active amino acid fermentation, if present, can produce short-chain fatty acids (e.g., butyrate and acetate) known to be utilized by heterotrophs in deep sediments (> 2 km depth) with nitrate or elemental sulfur as primary electron acceptors[22, 23]. Genes for glutamate dehydrogenase, glutamine synthetase and ammonia transporters were abundant in all metagenomes (Fig. 3b), indicating intracellular uptake and metabolic recycling of ammonia into non-essential amino acids.

Evidence for CO_2 fixation

Genes related to the 3HP/4HB pathway were encoded in both slope and bottom-axis metagenomes, and were highly transcribed in the bottom-axis sediments. The annotation of most of these genes to Thaumarchaeota implies that this taxon is among the major contributors to labile carbon in CD sediments in addition to Planctomycetes and NOB.

Other detected autotrophic pathways involved ten Nitrospinae and five Nitrospirae MAGs from both slope and trench axis sediments that encoded ATP-citrate lyase (*aclAB*) related to the reductive TCA pathway (rTCA). Higher abundance of genes involved in the rTCA pathway was observed in metagenomes from slope compared to bottom-axis metagenomes, that presented higher abundance of genes involved in the Wood–Ljungdahl (WL) pathway (Supplementary Fig. 14).

As mentioned, our data showed potential for autotrophic CO_2 fixation via the WL in the bottom-axis sediments. The *acsA* and *acsB* key genes identified in the MAGs were annotated to Planctomycetota, which suggests that Planctomycetota might conduct autotrophic carbon fixation in CD via the WL pathway using nitrite as an electron donor as previously described[24]. Bacteria can also produce acetate via carbon fixation pathways, and specifically via the WL pathway (reductive acetogenesis) [25]. Reductive acetogenesis requires the enzyme acetate kinase that is involved in the last step of acetate formation [26] and is encoded by the *ack* gene. *ack* was present together with the key WL genes in four Planctonomycetota MAGs while it was also affiliated with other bacterial phyla present in our metagenome and metatranscriptome data (Figs 3a-c). This suggests acetate production in CD sediments via reductive acetogenesis, the pta-ack pathway [26] and/or via fermentation, as previously discussed.

Nitrogen metabolism

The key genes of denitrification identified in our MAGs and metatranscriptomes were assigned to various bacterial taxa (Figs 3a-c). This supports that denitrification in hadal sediments is a modular process as described for other environments, where various bacterial groups can mediate different steps of the process [4, 27, 28]. The nitrate reductase gene *narG* was more abundant in bottom-axis compared to slope metagenomes (Supplementary Fig. 14, and "Geochemistry of Challenger Deep (CD) sediments"). Denitrification has been detected in both oxic and anoxic deep sediment layers in Mid-Ocean Ridge sediments[29], which indicates that denitrification, even at low rates[29], is an active nitrogen release process in the deep sediment column. Here we show a suite of denitrification genes (e.g., *narG*, *norB*, *nirK*, *nosZ*) in MAGs and metatranscriptomes at 10,911 m depth. We therefore suggest that denitrification in these hadal CD sediments is an active respiratory process for energy gain which agrees with previous studies[30]. However, the detection of *nosZ* gene in our "omic" data is reported for the first in hadal sediments, and indicates active the last step of denitrification and subsequent N_2 release.

Respiration of nitrogen-bearing compounds seems to be an essential process in CD sediments for energy gain which agrees with previous studies[30]. Nonetheless, our data show potential links between denitrification and chemoautotrophy as well as production of labile organic matter. Metatranscriptomics data from the bottom-axis sediment site T3L11 (10,908 m) showed active transcription of the ammonia monooxygenase gene (*amoA*), which implies nitrification coupled to autotrophic carbon fixation by AOA and NOB (Figs 3a-c and Supplementary Fig. 14). *amoA* was also the most abundant nitrification gene identified in the metagenomes and metatranscriptomes (Figs 3b, c, and Supplementary Fig. 14). Other genes involved in chemolithotrophic metabolisms included *nrxA* involved in nitrite oxidation that was encoded by 15 NOB MAGs. Phylogenetic

analyses showed protein divergence between NarG and NxrA from anammoxers, Nitrospina and Nitrospira (Supplementary Fig. 13).

Our knowledge of how anammoxers sustain anammox activity in hadal sediments is limited. Engström et al.,[31] suggested that anammox bacteria in deep sediments (2,800-3,100 m depth; Cascadia Basin) may rely on microbial dissimilatory nitrate reduction to ammonium (DNRA) and organic matter mineralization for ammonium supply. The *nrfA* or *nirB* gene was detected in our metagenomes (69 MAGs) (Fig. 5), which is consistent with previous studies of abyssal and hadal sediments (Yap Trench; 4,435–6,578 m depth)[32]. However, direct measurement of DNRA activity is required to estimate the contribution of the process in CD sediments, since studies also suggest negligible DNRA activity in nutrient-starved sediments collected below 4,000 m depth in Mid-Atlantic Ridge[29]. Anammox bacteria can also produce ammonium from nitrate reduction[33] and/or utilize alternative electron donors (e.g., formate, acetate, propionate), instead of ammonium, coupled with NO_3^- and NO_2^- for energy conservation[34, 35]. Detailed studies of anammox bacteria from oxygen minimum zones (OMZs) suggested that anammox can lead to production of ammonium by utilizing organic nitrogen substrates (e.g., urea and cyanate) that result from microbial degradation of dissolved organic matter[36]. Although OMZs cannot be compared to hadal CD sediments, and measurements of organic nitrogen substrates were beyond the scope of our study, we suggest that CD anammox bacteria may utilize/degrade organic N substrates that accumulate in the bottom-axis sediments for ammonium production (Fig. 5).

Finally, we detected MAGs (e.g., MAG B3T3L8), that contained denitrification genes and were abundant in the anoxic layers (> 18 cmbsf) of two sediment cores from the bottom-axis sites, similar to anammox MAGs. This indicates N_2 released from the hadal sediments by both processes. However, the contribution of each process in N_2 release needs to be further elucidated in CD

sediments using experimental incubations both in the laboratory and in situ as in Atacama and Kermadec Trenches [37].

Selenium metabolism

Diverse bacterial phyla are known to encode genes for selenate and/or selenite reduction for detoxification and/or energy gain, as well as genes for the synthesis of selenocysteine (Sec)[38]. Selenocysteine is a proteinogenic amino acid that can be utilized by both aerobic and anerobic bacteria; however comparative genomics showed that most bacterial lineages have lost the ability to use selenium over evolutionary time, making Sec utilization uncommon[39]. Expression of biosynthetic *sel* genes in the hadal microbes of the bottom-axis may indicate a niche-specific adaptation that allows microbes to utilize and biologically transform selenium into their proteome. The *sel* genes were possibly acquired via horizontal gene transfer[40].

Sulfur metabolism

Our metagenome and metatranscriptome data suggest that hydrogen sulfide is assimilated by CD microbes to synthesize sulfur-bearing amino acids like cysteine via the cysteine synthase gene *cysK* detected in half of our MAGs (~51%, Supplementary Data 7). *cysK* and other assimilatory sulfate reduction genes are reported for the first time in hadal realms. The wide presence of *cysK* suggests that sulfur-bearing amino acids can be essential to CD sediment microbes for basic metabolism (e.g., catabolism of cysteine provides thioester derivatives such as acetyl-CoA that can fuel Krebs cycle under aerobic respiration[41]), or as amino acid residues in proteins involved in heavy metal detoxification[42].

Our MAGs showed also high presence of genes associated with dissimilatory sulfate reduction (DSR) (Fig. 3a), which indicates sulfate reduction as an active process in CD sediments. However, only five MAGs affiliated to Proteobacteria (MAGs B43T3L14, B5T3L11, B60T3L14, B110T1L10 and B18T1L10) presented all genes for a complete DSR pathway. The sulfate reduction genes identified in CD sediments warrant future efforts for identifying their functional and structural diversity. Different microbial groups may share a homolog via horizontal gene transfer or may have evolved group-specific genes.

Supplementary Figures

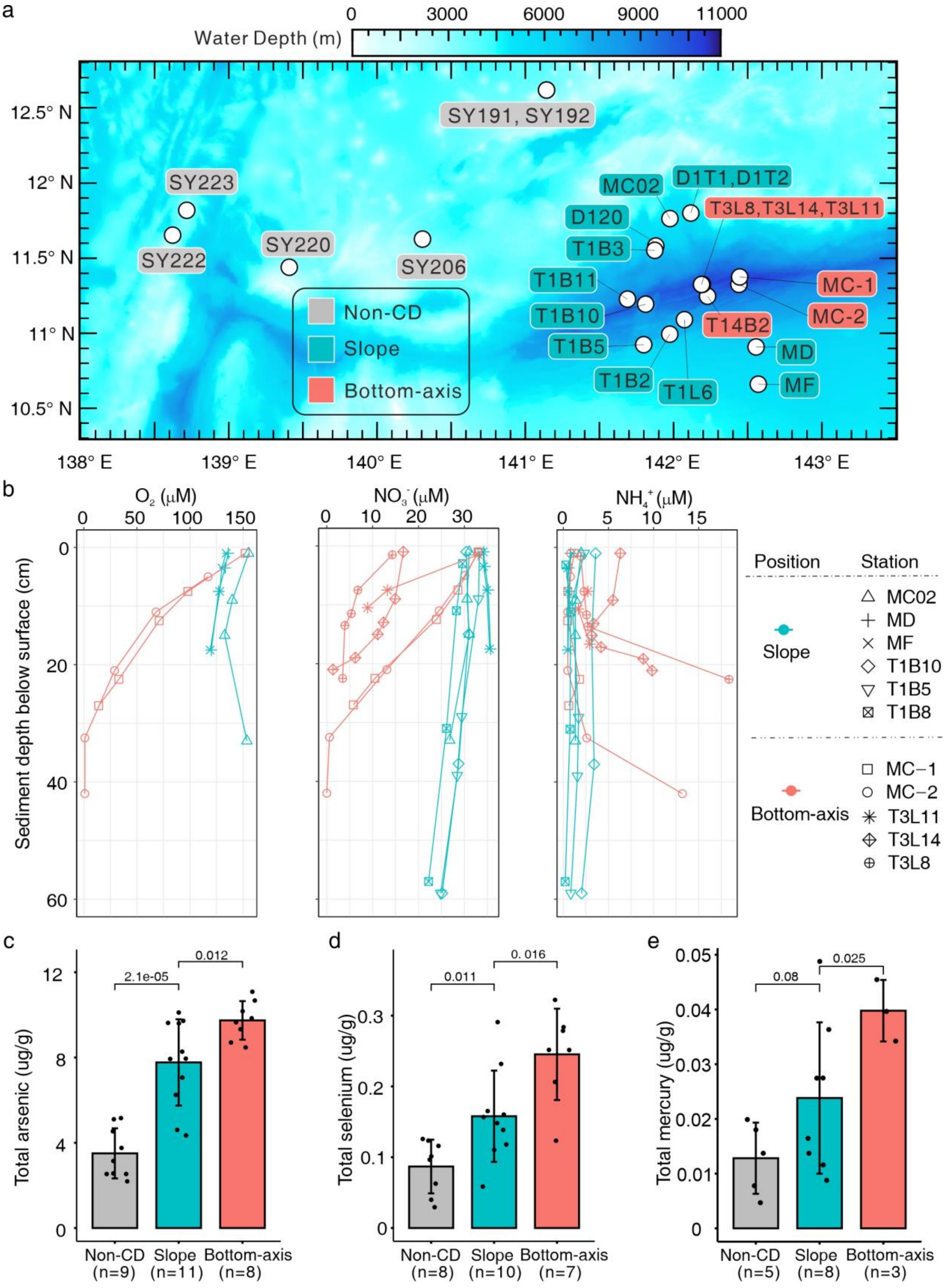

Supplementary Fig. 1 | Geochemistry data from sediments in Challenger Deep. a, Locations of samples. **b**, Oxygen (O_2), nitrate (NO_3^-) and ammonium (NH_4^+) concentrations in slope and bottom-axis sediments from the Challenger Deep (0-60 cm below seafloor; cmbsf). MF (5,183 m depth) and MD (6,067 m depth) are slope reference sites[43], ~69 km and ~88 km from the T1B5 slope site, respectively. MC-1 (10,901 m depth) and MC-2 (10,902 m depth) are bottom-axis reference sites[43], ~36 km from the bottom-axis site T3L11. **c**,**d,e** Total arsenic, selenium and mercury concentrations (μg/g of dry sediment) in 13 CD sites (5,400-10,911 m depth), and six nearby non-hadal reference sites (2,681-3,510 m depth). n represents the number of biologically independent samples. p values for pairwise comparisons were estimated using two-sided Wilcoxon test. Source data are provided as a Source Data file.

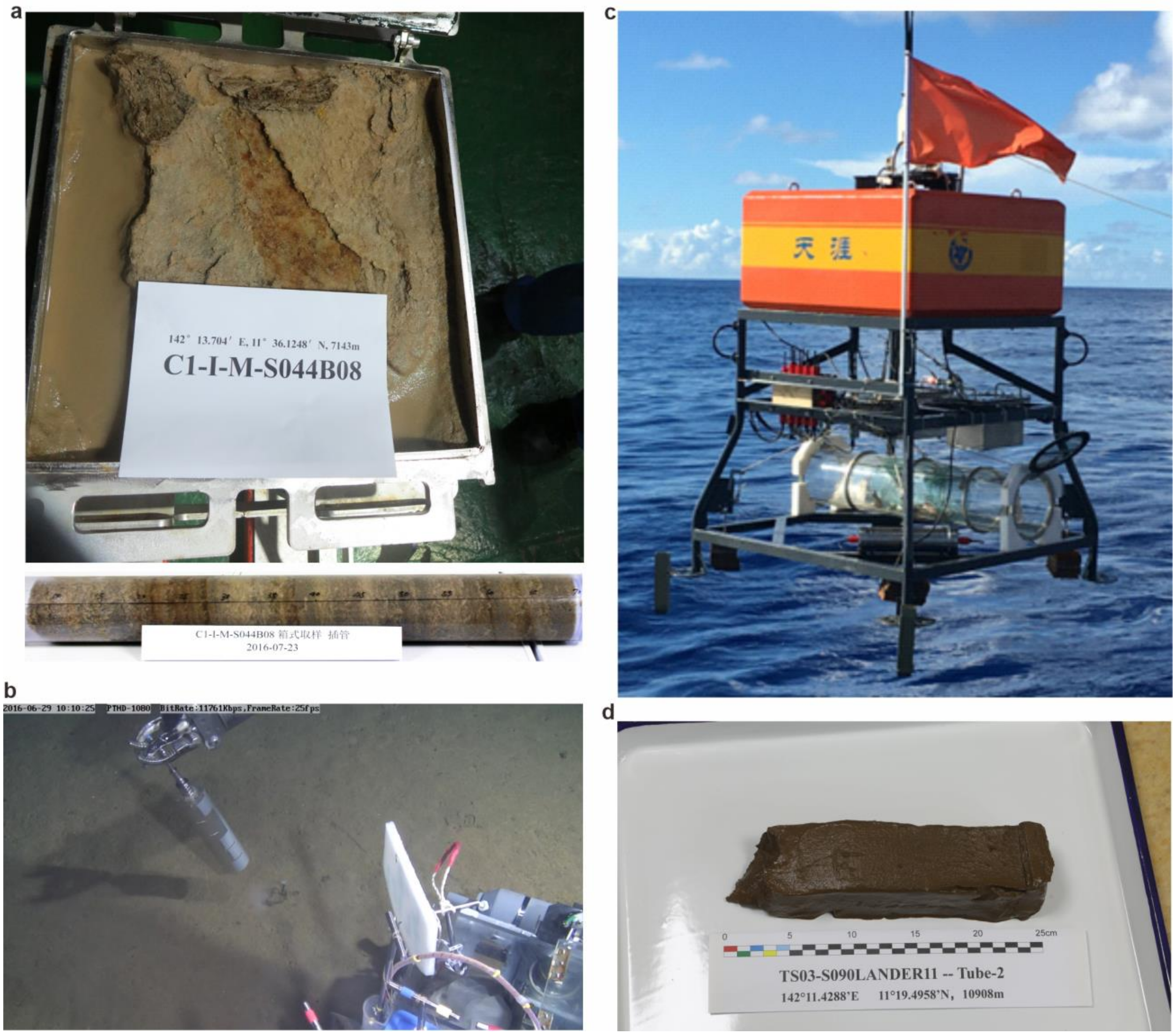

Supplementary Fig. 2 | Overview of sampling methods. Photos of three types of sampling methods: box core (**a**), push core from the submersible Dive121 at 5,500 m depth (**b**) and push core obtained by a lander (**c, d**). Both ends of the core sampler mounted on the feet of the lander (**c**) were tightly closed when the lander was lifted. The lander returned to the sea surface with the push cores in ~ 4 hours.

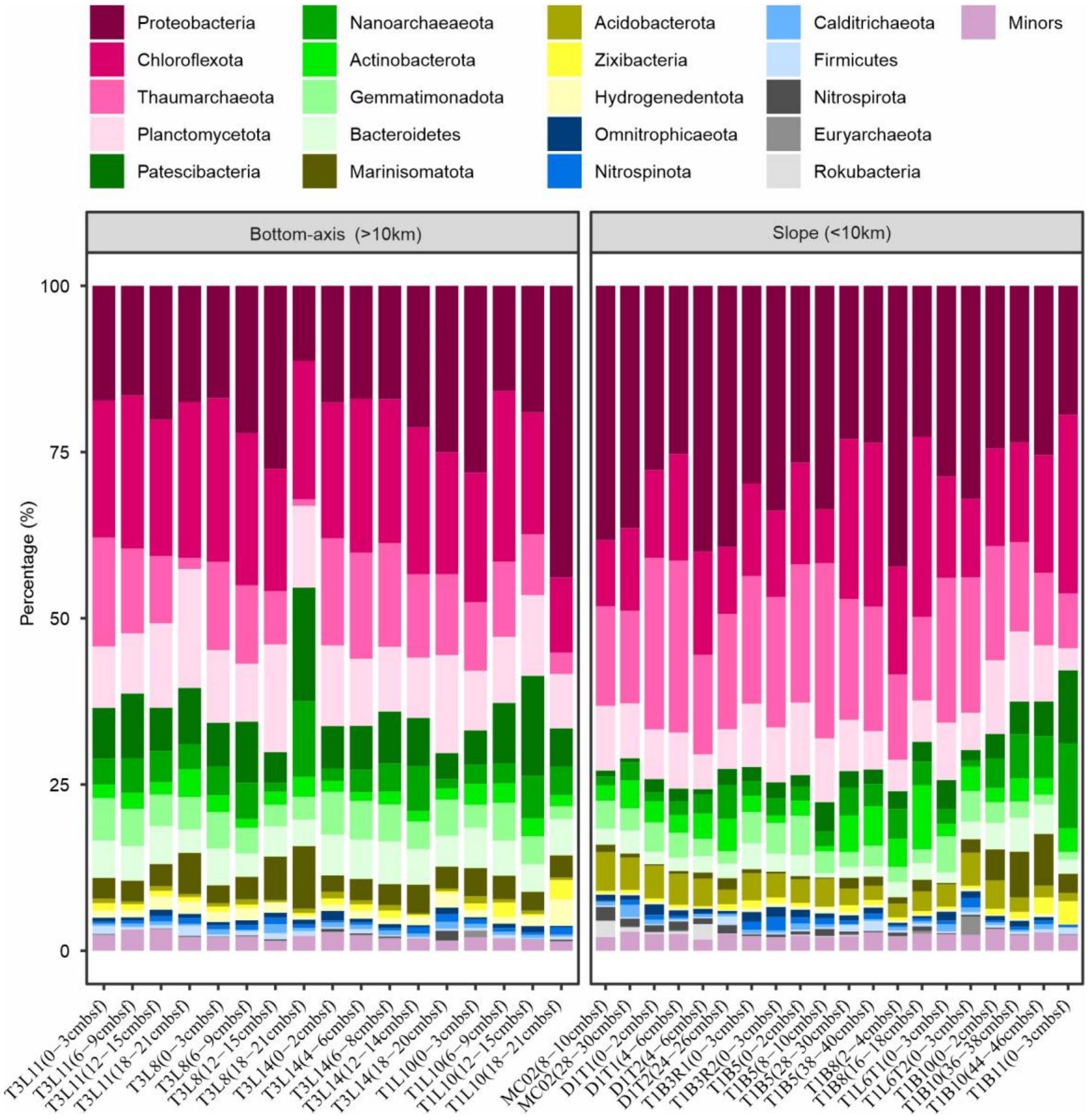

Supplementary Fig. 3 | Microbial community structure in Challenger Deep. Relative abundance of prokaryotes using 16S miTags at phylum level. Unclassified 16S miTags were not included in downstream analysis and phyla with low relative abundances (<1%) are merged. Source data are provided as a Source Data file.

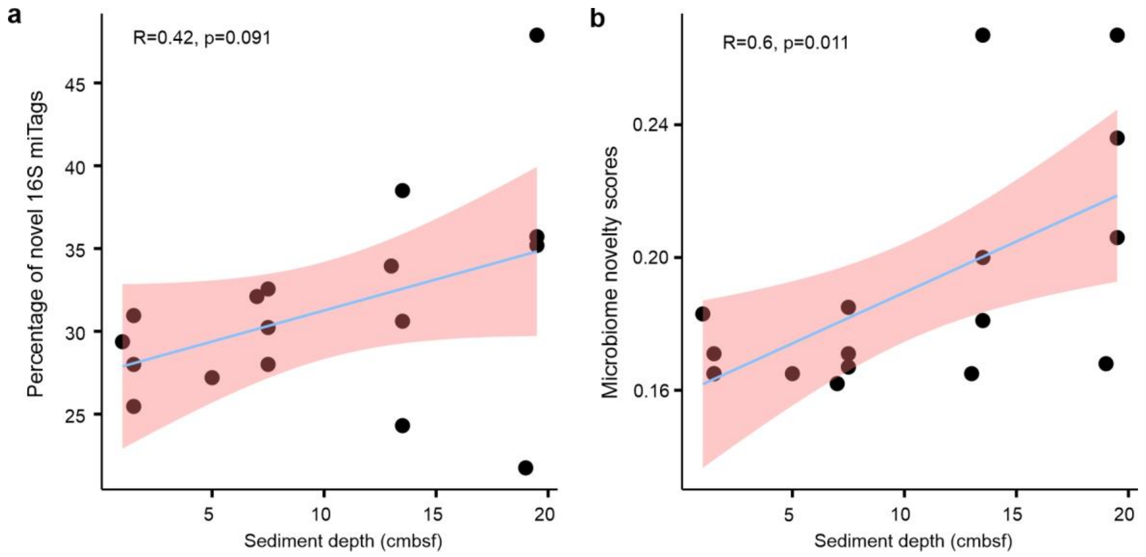

Supplementary Fig. 4 | Microbiome novelty in 17 metagenomes from bottom-axis sediments (T1L10, T3L11, T3L8, and T3L14; 0-21 cmbsf). a, Pearson's correlation analyses between the novel 16S miTags rates and the depth of the sampling site. **b**, Pearson's correlation analyses of the microbiome novelty with the sampling depth. p values after two-sided t test. The pink background represents the 95% confidence interval. Source data are provided as a Source Data file.

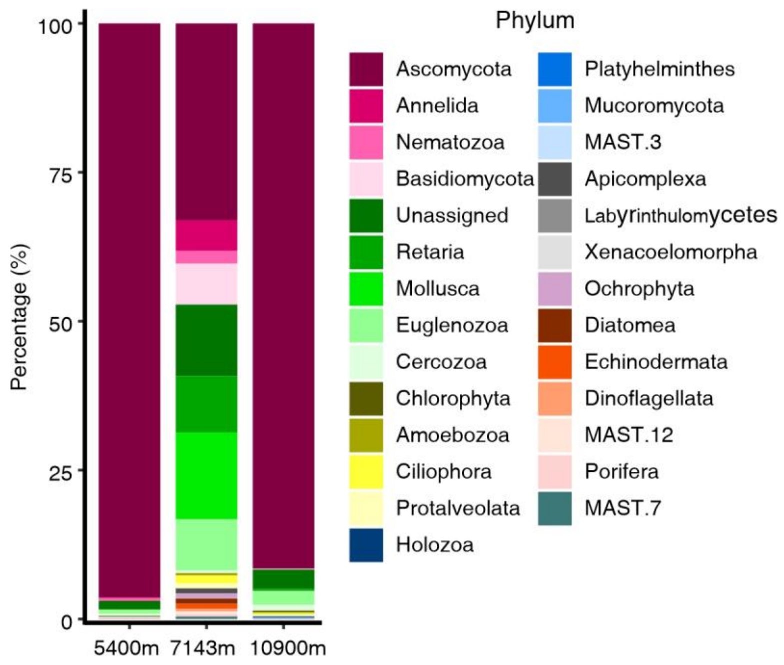

Supplementary Fig. 5 | Eukaryotic communities in sediment cores collected at slope and bottom-axis sites based on 18S miTags. MC02 (5,400m), T1B8 (7,143m), T1L10 (10,900m), T3L11 (10,908m), and T3L14 (10,911m). 18S miTag results obtained from sediment cores T1L10, T3L11 and T3L14 were merged. Taxonomic sorting of 18S miTags (>200) was against SILVA138 database. Source data are provided as a Source Data file.

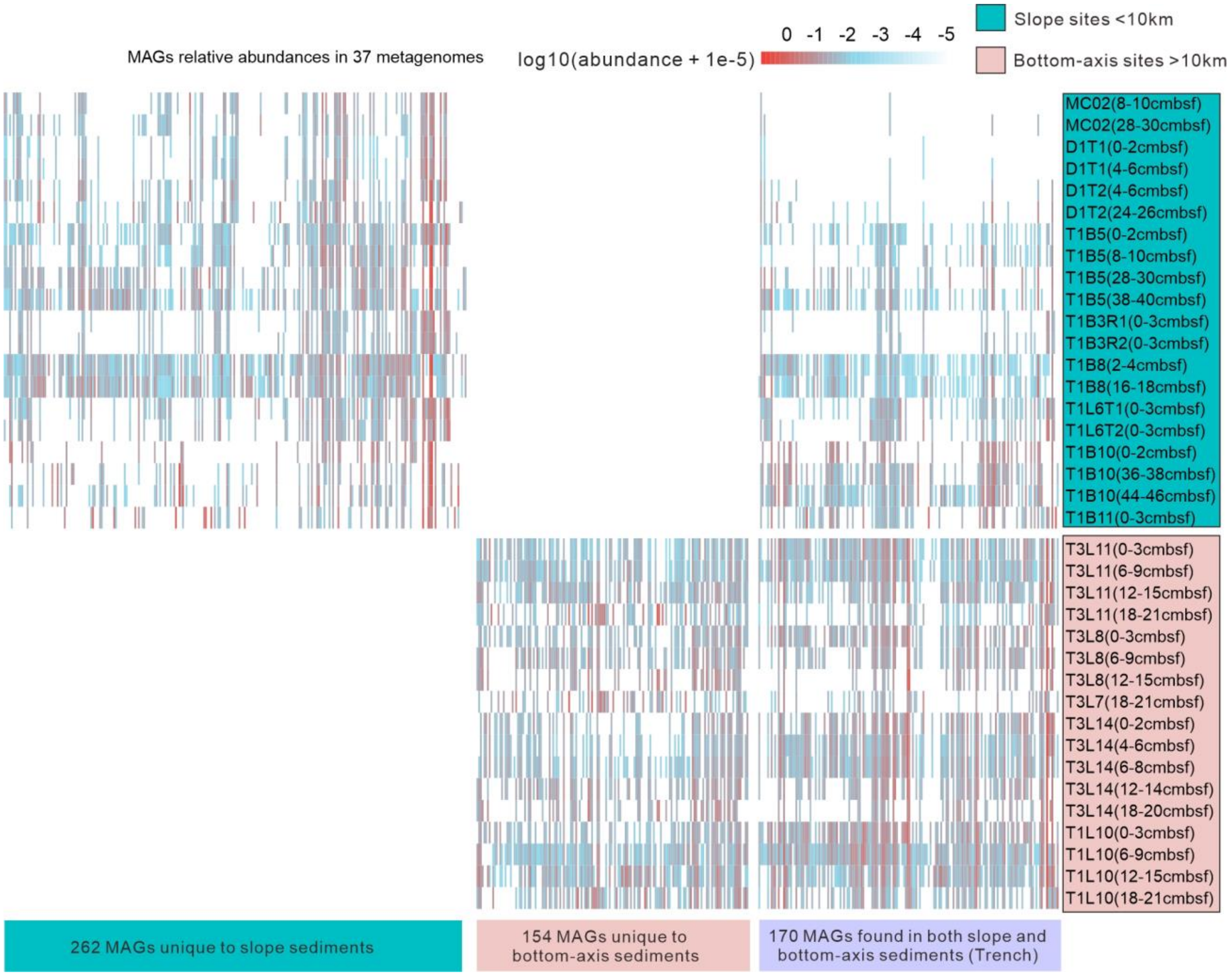

Supplementary Fig. 6 | Relative abundance of the MAGs in the 37 metagenomes. The relative abundances of each MAG were estimated using the CoverM and were transformed in the logarithmic scale of 10 (log10(abundance +1e-5)). The relative abundances of all 586 MAGs can be found in the Source Data file.

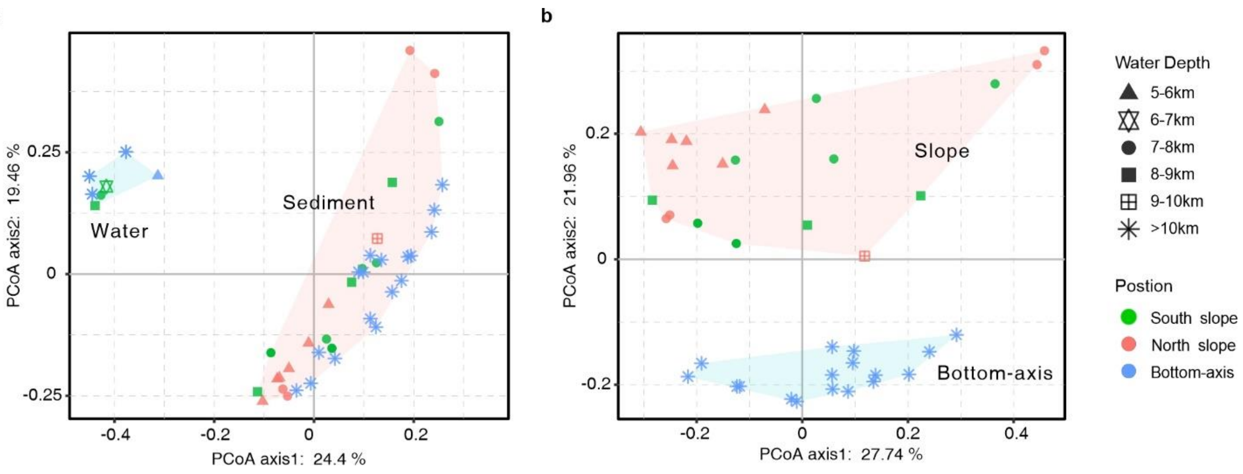

Supplementary Fig. 7 | PCoA analysis of CD prokaryotic communities using the relative abundance of 16S miTags data at species level. a, Bray-Curtis dissimilarity principal coordinate analyses (PCoA) of the prokaryotic communities in waters and sediments. **b,** Bray-Curtis dissimilarity principal coordinate analyses (PCoA) of the prokaryotic communities in sediments. **a** and **b** share the same legends. The metagenomic datasets for the hadal water samples were obtained from Gao et al. (2019)[44]. Source data are provided as a Source Data file.

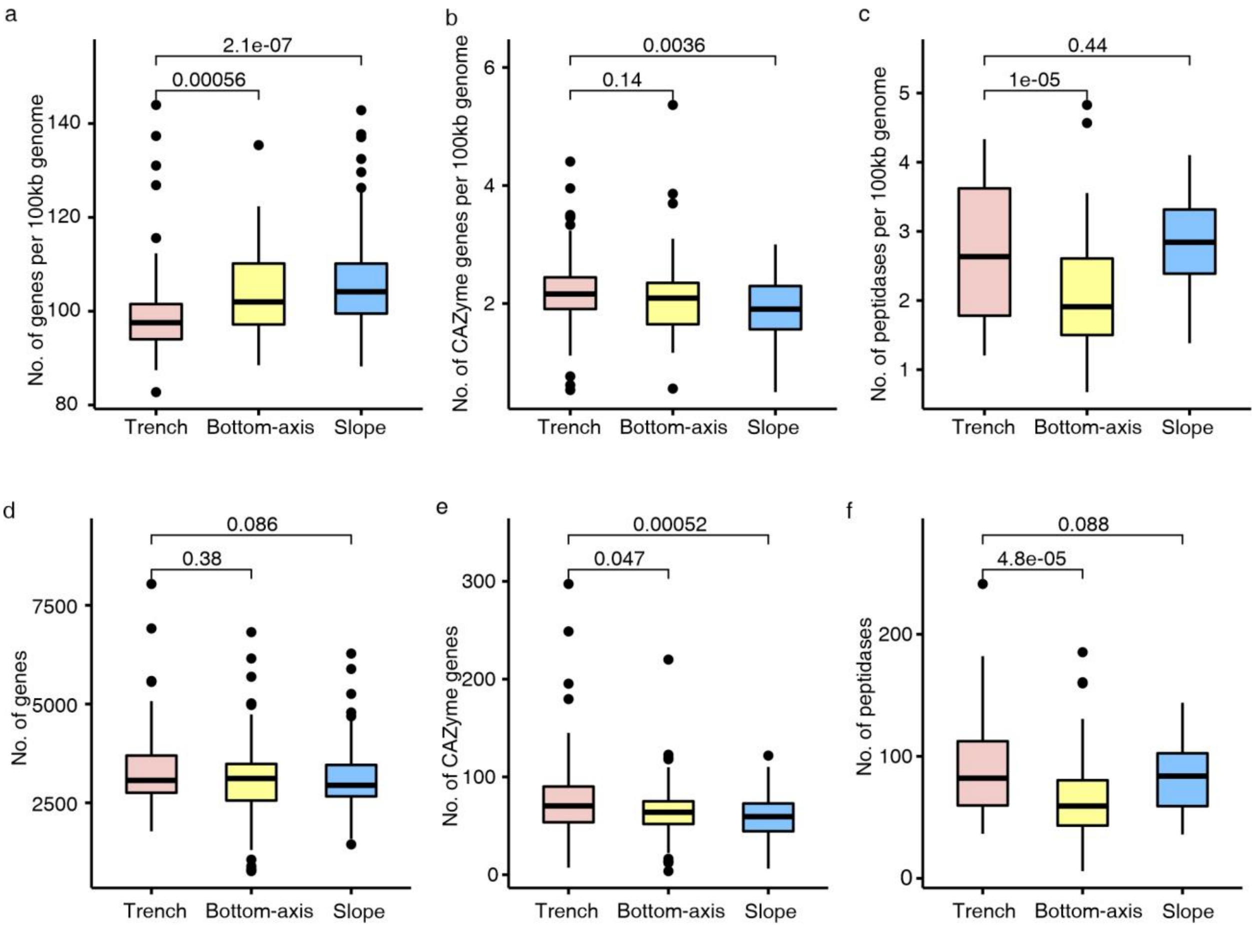

Supplementary Fig. 8 | Gene density and number of CAZyme- and peptidase-coding genes in MAGs. Gene density was plotted for the 80 most prevalent MAGs in bottom-axis (Bottom), slope (Slope) and slope and bottom-axis sediments (Trench) MAGs (**a,d**). Number of genes coding for CAZymes, (**b,e**) and peptidases (**c,f**) in the 80 most prevalent MAGs. Two-sided t-test was performed to compare the gene numbers. p values are shown in the figures. In boxplot, center lines indicate median values. The lower and upper bounds represent 25th and 75th percentiles, respectively. The lower/upper whiskers represent minima/maxima no further than 1.5 times interquartile range from the hinge, and the points falling outside of the whiskers represent the outliers. Source data are provided as a Source Data file.

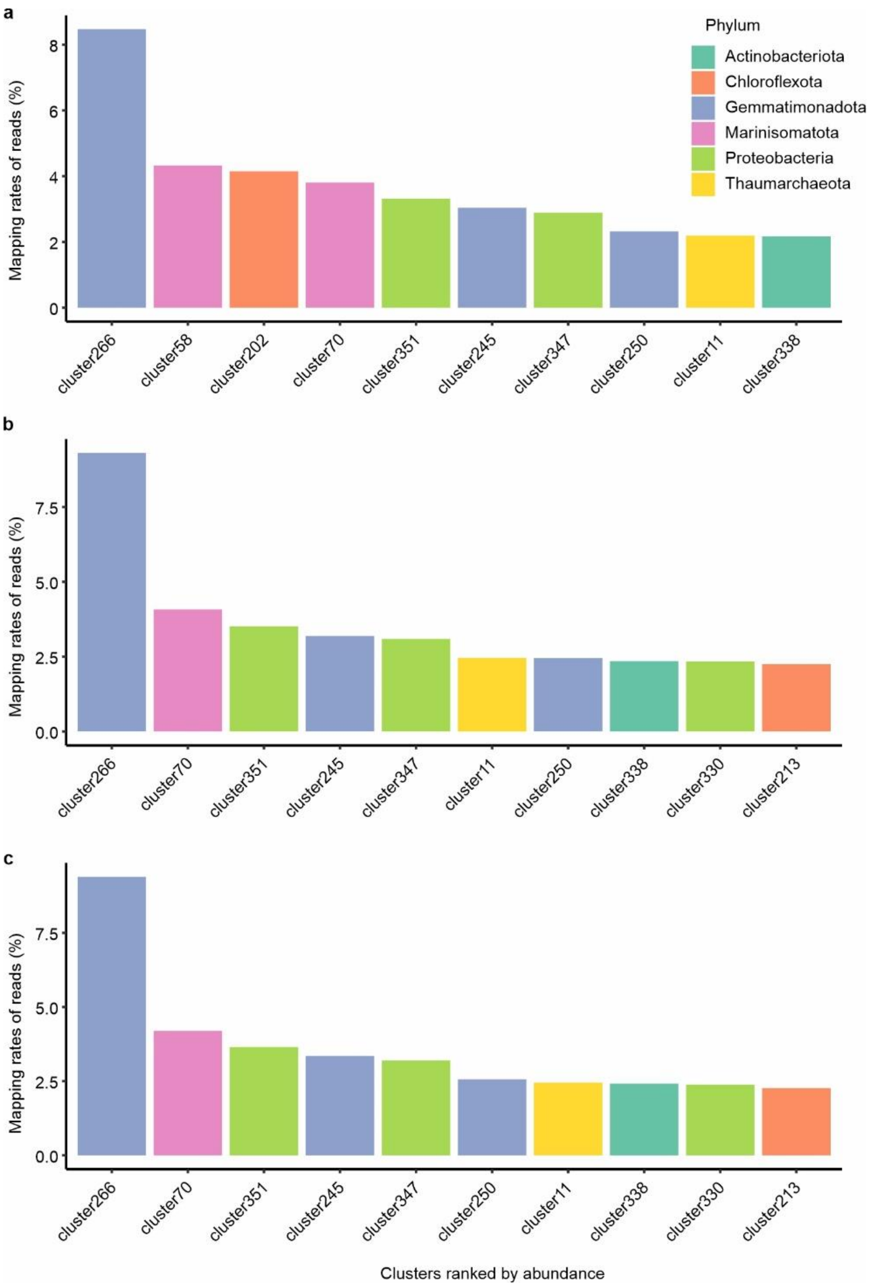

Supplementary Fig. 9 | 10 most abundant taxa clusters in the bottom-axis metatranscriptome libraries. Taxa clusters are composed of MAGs grouped at 95% Average Nucleotide Identity (ANI). The abundance was calculated by dividing the number of transcriptome reads mapped to genes of each cluster by the sum of the total metatranscriptome reads mapped to all genes of the MAGs (**a,** 6-9 cmbsf; **b,** 12-15 cmbsf; **c,** 18-21 cmbsf). Source data are provided as a Source Data file.

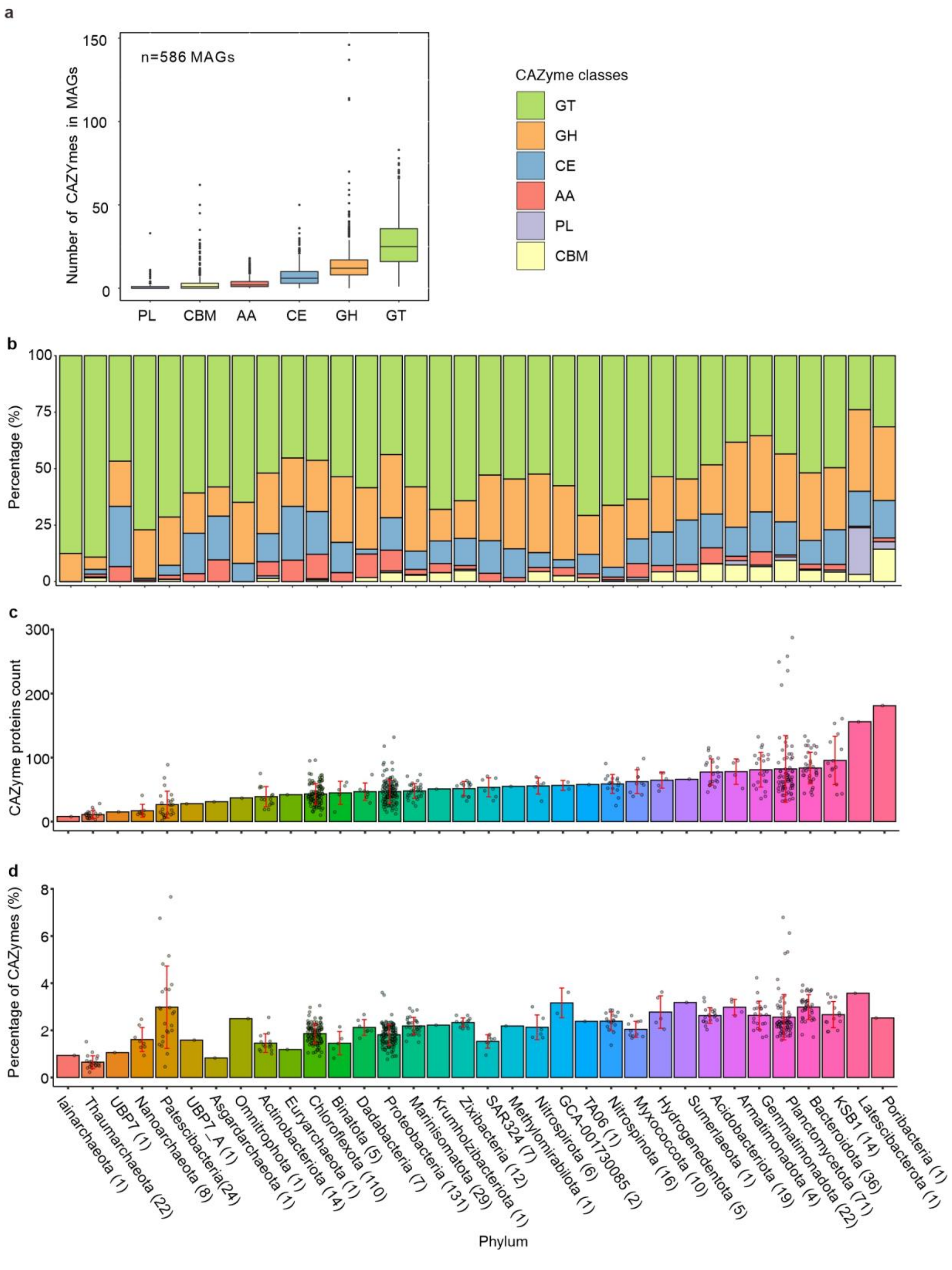

Supplementary Fig. 10 | Taxonomic and functional distribution of CAZymes in slope and bottom-axis CD MAGs. a, Number of CAZymes identified in CD MAGs (n=586) as classified

in six CAZyme classes. In boxplot, center lines indicate median values. The lower and upper bounds represent 25th and 75th percentiles, respectively. The lower/upper whiskers represent minima/maxima no further than 1.5 times interquartile range from the hinge, and the points falling outside of the whiskers represent the outliers. **b**, Presence of different CAZyme classes in various phyla (**a** and **b** share the same color legends for CAZyme classes). **c**, CAZyme protein counts in the 34 CD phyla. **d**, Presence of predicted CAZymes for each phylum. Error bars show mean ± SD (c, d). Numbers in parentheses indicate the number of genomes assigned to each phylum. AA: Auxiliary Activities; CBM: Carbohydrate-Binding Modules; CE: Carbohydrate Esterases; GH: Glycoside Hydrolases; GT: Glycosyl Transferases; PL: Polysaccharide Lyases. Source data are provided as a Source Data file.

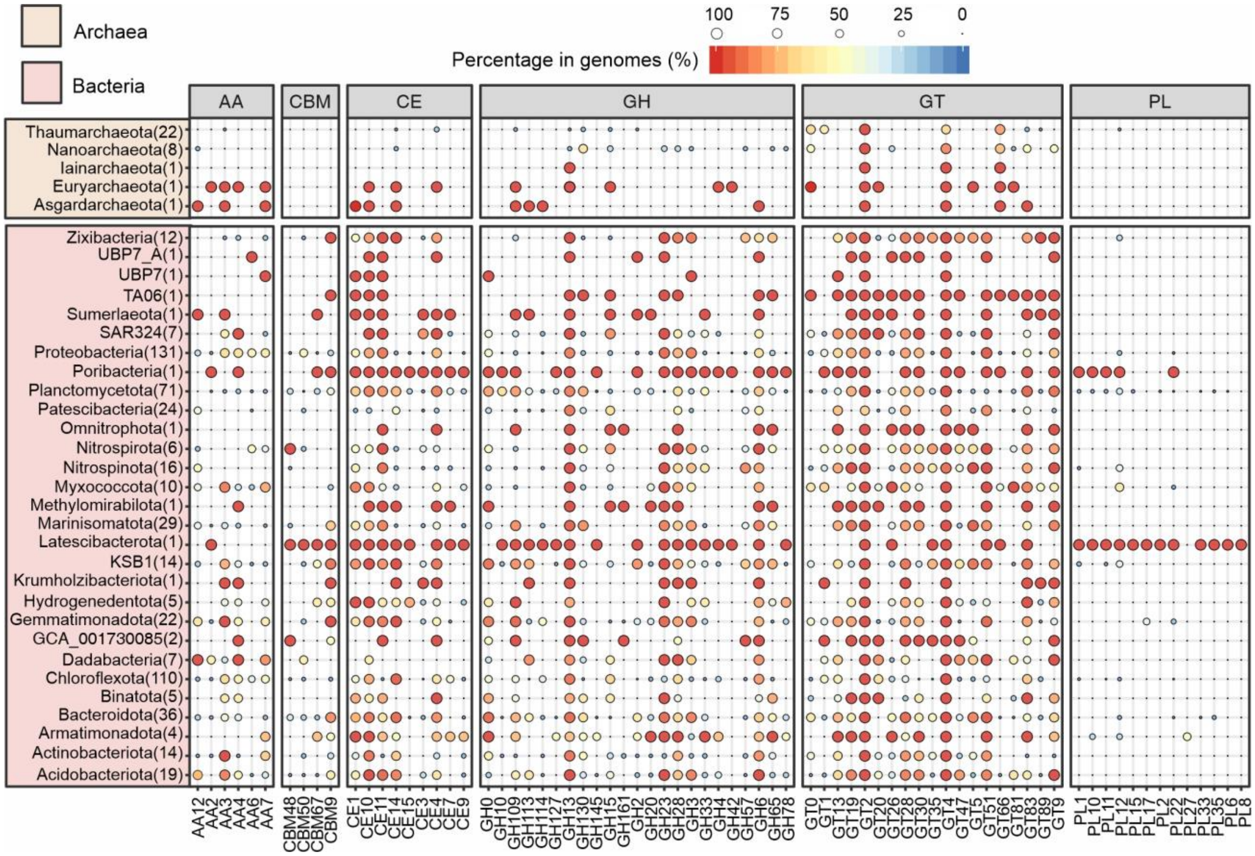

Supplementary Fig. 11 | Percentage of MAGs that contained CAZyme families. AA: Auxiliary Activities; CBM: Carbohydrate-Binding Modules; CE: Carbohydrate Esterases; GH: Glycoside Hydrolases; GT: Glycosyl Transferases; PL: Polysaccharide Lyases. The number of MAGs per phylum is shown in parentheses. Source data are provided as a Source Data file.

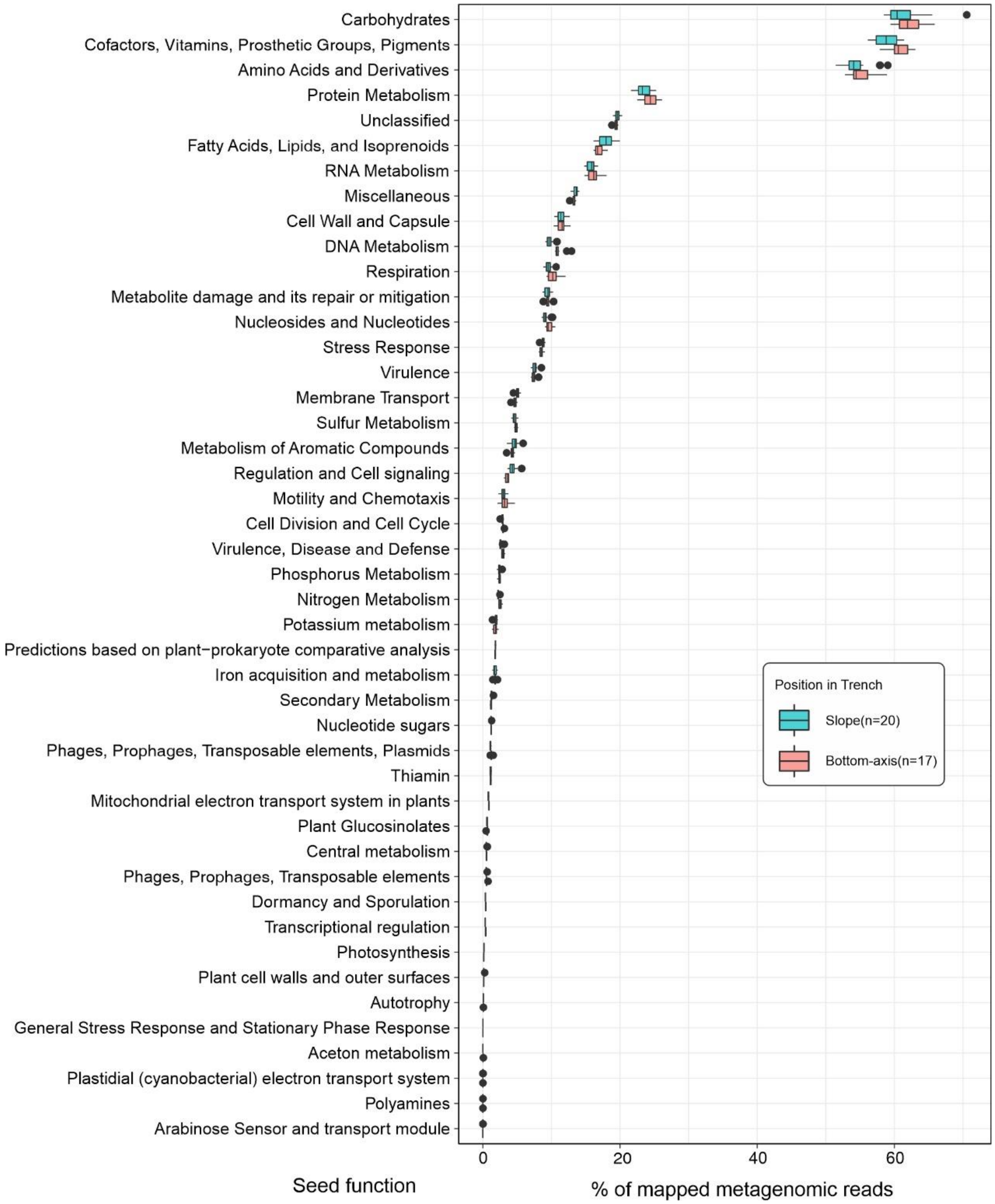

Supplementary Fig. 12 | Boxplot showing relative abundance of SEED functions. The functional categories of the reads in the metagenomes across the bottom-axis and slope CD sediments were searched using BLASTx against the GenBank NR database and parsed with the SEED database. In boxplot, center lines indicate median values. The lower and upper bounds represent 25th and 75th percentiles, respectively. The lower/upper whiskers represent

minima/maxima no further than 1.5 times interquartile range from the hinge, and the points falling outside of the whiskers represent the outliers. Source data are provided as a Source Data file.

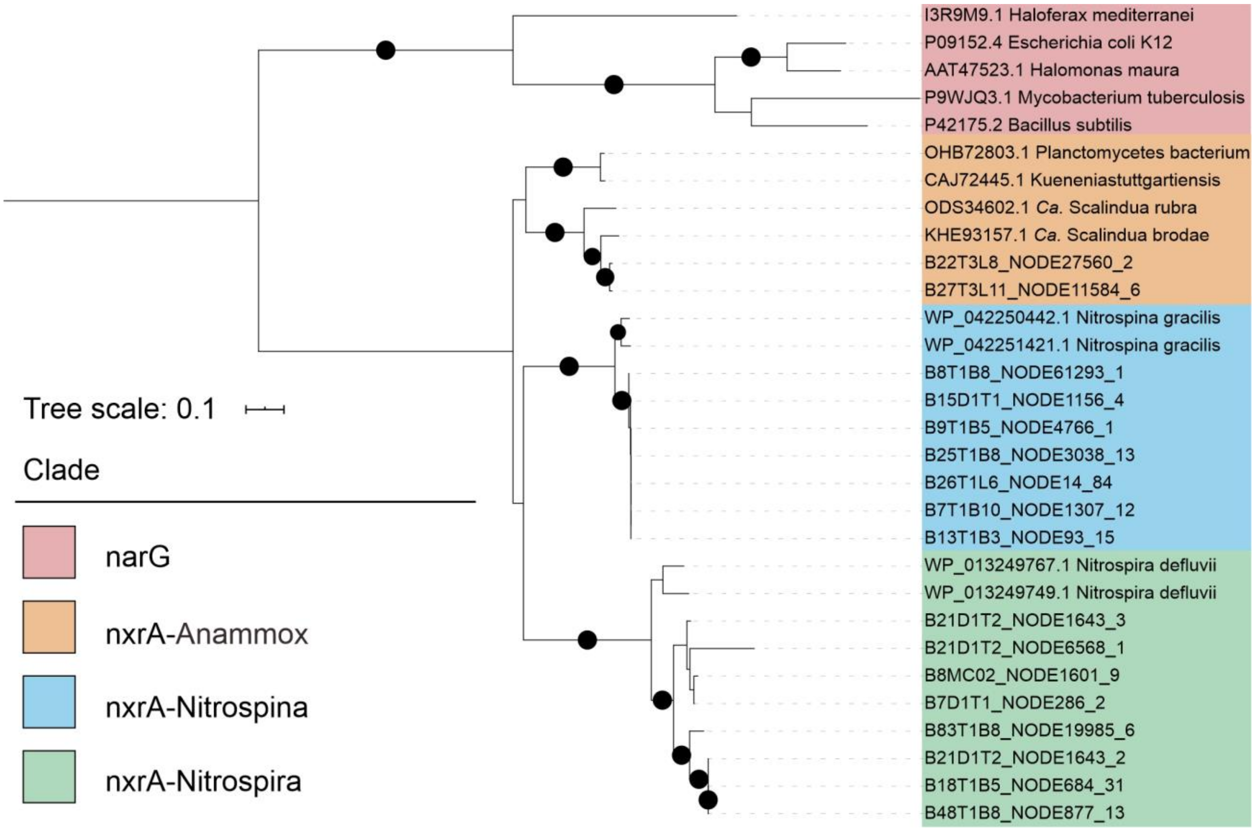

Supplementary Fig. 13 | Maximum-likelihood phylogenetic tree of anammox, *Nitrospira* and *Nitrospina* NxrA protein sequences. Bootstrap values (1,000 replicates) ≥ 80% are indicated by black dots.

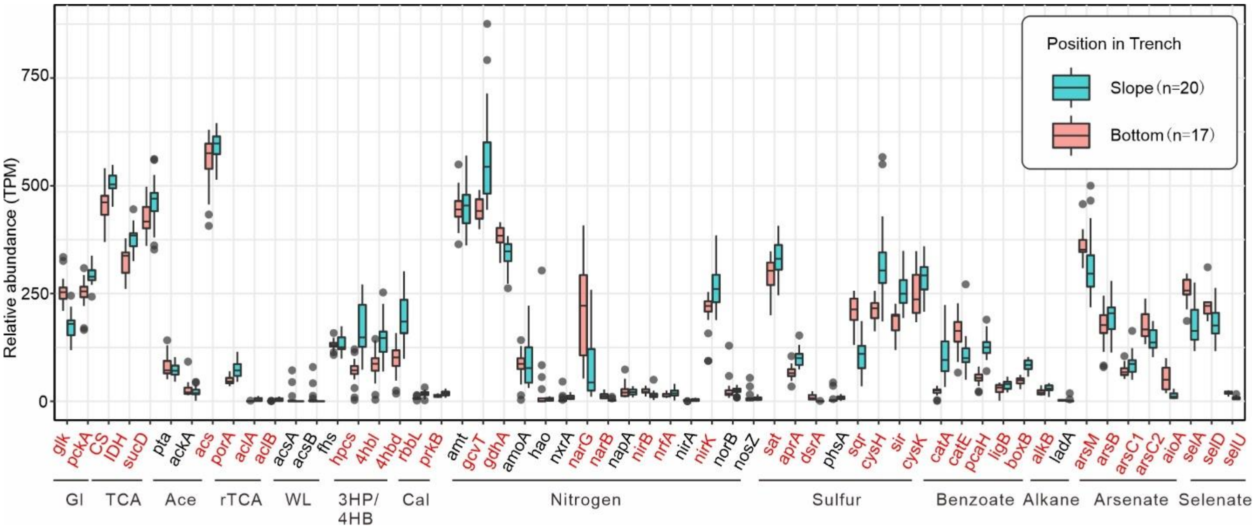

Supplementary Fig. 14 | Relative abundance of genes involved in nitrogen, sulfur and carbon cycling, CO_2 fixation, benzoate degradation, arsenate and selenate metabolisms from slope (n=20) and bottom-axis (n=17) sediment metagenomes. Genes in red were significantly different between the slope and bottom-axis metagenomes (two-sided t-test, $p<0.05$). In boxplot, center lines indicate median values. The lower and upper bounds represent 25th and 75th percentiles, respectively. The lower/upper whiskers represent minima/maxima no further than 1.5 times interquartile range from the hinge, and the points falling outside of the whiskers represent the outliers. Gl: glycolysis and gluconeogenesis, TCA: tricarboxylic acid cycle, Ace: acetate metabolism, rTCA: reductive tricarboxylic acid cycle, WL: Wood-Ljungdahl pathway, 3HP/4HB: 3-hydroxypropionate/4-hydroxybutyrate cycle, Cal: Calvin cycle. TPM: transcripts per million. Relative abundance of genes can be found in the Source Data file.

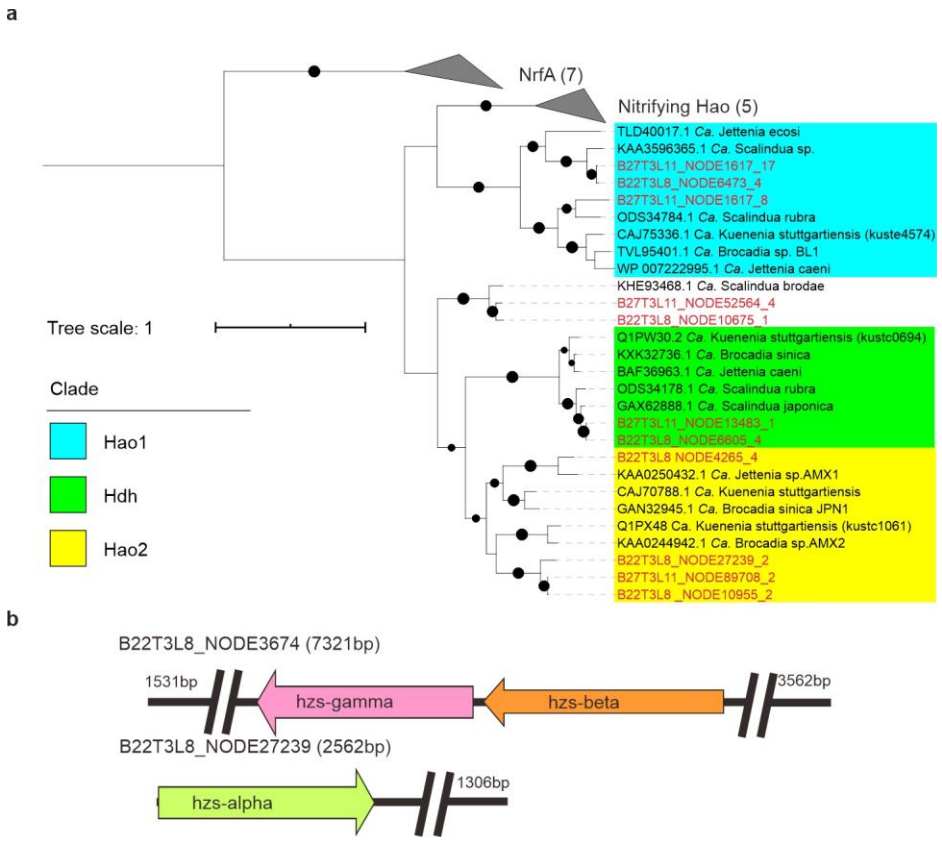

Supplementary Fig. 15 | Anammox proteins in deeper (> 15 cmbsf) bottom-axis sediments. **a,** Maximum-likelihood phylogenetic tree of the Hao-like proteins identified in the two recovered anammox CD MAGs (red), with published Hao-like proteins black. Bootstrap values (1,000 replicates) ≥ 80% are shown in black dots. Background colors indicate different clades of Hao-like proteins. **b,** Genes that encode the three subunits of hydrazine synthase (Hzs) in B22T3L8.

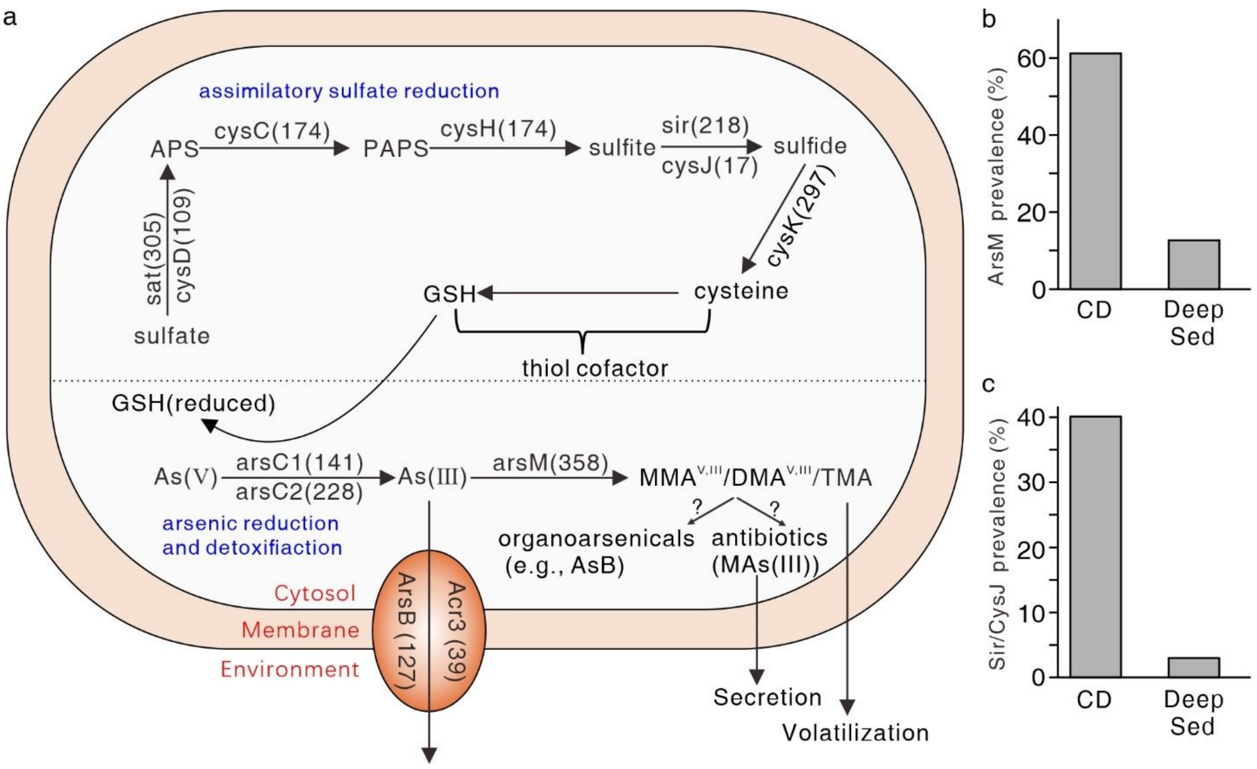

Supplementary Fig. 16 | Arsenic biotransformation in the Challenger Deep bottom-axis sediment. a, assimilatory sulfate, and arsenate reductions mediated by CD microbes (the number of MAGs containing the genes is in parentheses). **b, c,** prevalence of *arsM* and *sir* in MAGs from CD and deep-sea sediments (Guaymas Basin hydrothermal sediments)[4].

Competing interests

The authors declare no competing interests.

Supplementary References

1. Liu S, Peng X. Organic matter diagenesis in hadal setting: Insights from the pore-water geochemistry of the Mariana Trench sediments. *Deep Sea Res I* **147**, 22-31 (2019).

2. Glud RN*, et al.* High rates of microbial carbon turnover in sediments in the deepest oceanic trench on Earth. *Nat Geosci* **6**, 284-288 (2013).

3. Luo M, Gieskes J, Chen LY, Shi XF, Chen DF. Provenances, distribution, and accumulation of organic matter in the southern Mariana Trench rim and slope: Implication for carbon cycle and burial in hadal trenches. *Mar Geol* **386**, 98-106 (2017).

4. Dombrowski N, Teske AP, Baker BJ. Expansive microbial metabolic versatility and biodiversity in dynamic Guaymas Basin hydrothermal sediments. *Nat Commun* **9**, 4999 (2018).

5. Miller IJ, Weyna TR, Fong SS, Lim-Fong GE, Kwan JC. Single sample resolution of rare microbial dark matter in a marine invertebrate metagenome. *Sci Rep* **6**, 34362 (2016).

6. Peoples LM*, et al.* Microbial community diversity within sediments from two geographically separated hadal trenches. *Front Microbiol* **10**, 347 (2019).

7. Overbeek R*, et al.* The subsystems approach to genome annotation and its use in the project to annotate 1000 genomes. *Nucleic Acids Res* **33**, 5691-5702 (2005).

8. Huang L*, et al.* dbCAN-seq: a database of carbohydrate-active enzyme (CAZyme) sequence and annotation. *Nucleic Acids Res* **46**, D516-D521 (2018).

9. Breton C, Snajdrova L, Jeanneau C, Koca J, Imberty A. Structures and mechanisms of glycosyltransferases. *Glycobiology* **16**, 29R-37R (2006).

10. Coutinho PM, Deleury E, Davies GJ, Henrissat B. An evolving hierarchical family classification for glycosyltransferases. *J Mol Biol* **328**, 307-317 (2003).

11. Nakamura AM, Nascimento AS, Polikarpov I. Structural diversity of carbohydrate esterases. *Biotechnol Res Inn* **1**, 35-51 (2017).

12. Ganesh Kumar A, Mathew NC, Sujitha K, Kirubagaran R, Dharani G. Genome analysis of deep sea piezotolerant Nesiotobacter exalbescens COD22 and toluene degradation studies under high pressure condition. *Sci Rep* **9**, 18724 (2019).

13. Yakovlieva L, Walvoort MTC. Processivity in Bacterial Glycosyltransferases. *ACS Chem Biol* **15**, 3-16 (2020).

14. Muller AU, Weber-Ban E. The bacterial proteasome at the core of diverse degradation pathways. *Front Mol Biosci* **6**, 23 (2019).

15. Pearce MJ, Mintseris J, Ferreyra J, Gygi SP, Darwin KH. Ubiquitin-like protein involved in the proteasome pathway of *Mycobacterium tuberculosis*. *Science* **322**, 1104-1107 (2008).

16. Elharar Y, Roth Z, Hecht N, Rotkopf R, Khalaila I, Gur E. Posttranslational regulation of coordinated enzyme activities in the Pup-proteasome system. *Proc Natl Acad Sci USA* **113**, E1605-E1614 (2016).

17. Phale PS, Malhotra H, Shah BA. Chapter One - Degradation strategies and associated regulatory mechanisms/features for aromatic compound metabolism in bacteria. In: *Advances in Applied Microbiology* (eds Gadd GM, Sariaslani S). Academic Press (2020).

18. Valderrama JA, Durante-Rodríguez G, Blázquez B, García JL, Carmona M, Díaz E. Bacterial degradation of benzoate cross-regulation between aerobic and anaerobic pathways. *J Biol Chem* **287**, 10494-10508 (2012).

19. Kuntze K, Vogt C, Richnow HH, Boll M. Combined application of PCR-based functional assays for the detection of aromatic-compound-degrading anaerobes. *Appl Environ Microb* **77**, 5056-5061 (2011).

20. Liu J*, et al.* Proliferation of hydrocarbon-degrading microbes at the bottom of the Mariana Trench. *Microbiome* **7**, 47 (2019).

21. Sauer U, Eikmanns BJ. The PEP-pyruvate-oxaloacetate node as the switch point for carbon flux distribution in bacteria. *FEMS Microbiol Rev* **29**, 765-794 (2005).

22. Teske A, Brinkhoff T, Muyzer G, Moser DP, Rethmeier J, Jannasch HW. Diversity of thiosulfate-oxidizing bacteria from marine sediments and hydrothermal vents. *Appl Environ Microbiol* **66**, 3125-3133 (2000).

23. Takaki Y*, et al.* Bacterial lifestyle in a deep-sea hydrothermal vent chimney revealed by the genome sequence of the thermophilic bacterium *Deferribacter desulfuricans* SSM1. *DNA Res* **17**, 123-137 (2010).

24. Strous M*, et al.* Deciphering the evolution and metabolism of an anammox bacterium from a community genome. *Nature* **440**, 790-794 (2006).

25. Ragsdale SW, Pierce E. Acetogenesis and the Wood–Ljungdahl pathway of CO_2 fixation. *Biochim Biophys Acta* **1784**, 1873-1898 (2008).

26. Wolfe AJ. The acetate switch. *Microbiol Mol Biol Rev* **69**, 12-50 (2005).

27. Graf DR, Jones CM, Hallin S. Intergenomic comparisons highlight modularity of the denitrification pathway and underpin the importance of community structure for N_2O emissions. *Plos One* **9**, e114118 (2014).

28. Dalsgaard T*, et al.* Oxygen at nanomolar levels reversibly suppresses process rates and gene expression in anammox and denitrification in the oxygen minimum zone off Northern Chile. *mBio* **5**, e01966-01914 (2014).

29. Zhao R, Hannisdal B, Mogollon JM, Jørgensen SL. Nitrifier abundance and diversity peak at deep redox transition zones. *Sci Rep* **9**, 8633 (2019).

30. Nunoura T*, et al.* Microbial diversity in sediments from the bottom of the Challenger Deep, the Mariana Trench. *Microbes Environ* **33**, 186-194 (2018).

31. Engström P, Penton CR, Devola AH. Anaerobic ammonium oxidation in deep-sea sediments off the Washington margin. *Limnol Oceanogr* **54**, 1643-1652 (2009).

32. Zhang X, Xu W, Liu Y, Cai M, Luo Z, Li M. Metagenomics reveals microbial diversity and metabolic potentials of seawater and surface sediment from a hadal biosphere at the Yap Trench. *Front Microbiol* **9**, 2402 (2018).

33. Kartal B*, et al.* Anammox bacteria disguised as denitrifiers: nitrate reduction to dinitrogen gas via nitrite and ammonium. *Environ Microbiol* **9**, 635-642 (2007).

34. Lawson CE*, et al.* Autotrophic and mixotrophic metabolism of an anammox bacterium revealed by in vivo ^{13}C and ^{2}H metabolic network mapping. *ISME J*, (2020).

35. Güven D*, et al.* Propionate oxidation by and methanol inhibition of anaerobic ammonium-oxidizing bacteria. *Appl Environ Microb* **71**, 1066-1071 (2005).

36. Ganesh S*, et al.* Single cell genomic and transcriptomic evidence for the use of alternative nitrogen substrates by anammox bacteria. *ISME J* **12**, 2706-2722 (2018).

37. Thamdrup B*, et al.* Anammox bacteria drive fixed nitrogen loss in hadal trench sediments. *Proc Natl Acad Sci USA* **118**, e2104529118 (2021).

38. Stolz JF, Basu P, Santini JM, Oremland RS. Arsenic and selenium in microbial metabolism. *Annu Rev Microbiol* **60**, 107-130 (2006).

39. Peng T, Lin J, Xu Y-Z, Zhang Y. Comparative genomics reveals new evolutionary and ecological patterns of selenium utilization in bacteria. *ISME J* **10**, 2048-2059 (2016).

40. Zhang Y, Romero H, Salinas G, Gladyshev VN. Dynamic evolution of selenocysteine utilization in bacteria: a balance between selenoprotein loss and evolution of selenocysteine from redox active cysteine residues. *Genome Biol* **7**, R94 (2006).

41. Tsuchiya Y*, et al.* Protein CoAlation and antioxidant function of coenzyme A in prokaryotic cells. *Biochem J* **475**, 1909-1937 (2018).

42. Yang HC, Rosen BP. New mechanisms of bacterial arsenic resistance. *Biochem J* **39**, 5-13 (2016).

43. Hiraoka S*, et al.* Microbial community and geochemical analyses of trans-trench sediments for understanding the roles of hadal environments. *ISME J* **14**, 740–756 (2020).

44. Gao ZM*, et al.* In situ meta-omic insights into the community compositions and ecological roles of hadal microbes in the Mariana Trench. *Environ Microbiol* **21**, 4092-4108 (2019).
