# Supplementary material for: Microbiomes in the Challenger Deep slope and bottom-axis sediments
Source: Nat Commun. 2022 Mar 21;13:1515. doi: 10.1038/s41467-022-29144-4 (PMC8938466; doi:10.1038/s41467-022-29144-4)
Supplement: Supplementary file 3 — Description of Additional Supplementary Information [file 41467_2022_29144_MOESM3_ESM.docx]

**Description of Additional Supplementary Files**

**File Name:** Supplementary Data 1

**Description:** Geochemistry data (NO_3_^-^, NH_4_^+^, O_2_, TOC, TON) and heavy metal concentrations (arsenic, selenium, mercury) of slope and bottom-axis CD sediments. Sheet1: Porewater nutrients and organic geochemistry data. Sheet2: Concentration of heavy metals in 13 CD sites and six nearby non-hadal reference sites.

**File Name:** Supplementary Data 2

**Description:** The sampling sites and methods (box core, push core and push core obtained by a lander) used for collecting sediment cores across the CD trench.

**File Name:** Supplementary Data 3

**Description:** Overview of the metagenomes and metatranscriptomes generated from the slope and bottom-axis CD sediments.

**File Name:** Supplementary Data 4

**Description:** Overview of the prokaryotic MAGs identified in this study (n=586).

**File Name:** Supplementary Data 5

**Description:** Clusters of the CD MAGs at species level (ANI>95%).

**File Name:** Supplementary Data 6

**Description:** Summary of the annotated CAZymes in reconstructed CD sediment MAGs.

**File Name:** Supplementary Data 7

**Description:** Summary of the KEGG annotations identified in reconstructed CD sediment MAGs.

**File Name:** Supplementary Data 8

**Description:** Reference genomes used in the phylogenetic analysis of the CD sediment MAGs.

**File Name:** Supplementary Data 9

**Description:** Overview of the phylogenetic marker genes used in the phylogenetic analysis of the CD sediment MAGs.
