# Supplementary material for: Microbiomes in the Challenger Deep slope and bottom-axis sediments
Source: Nat Commun. 2022 Mar 21;13:1515. doi: 10.1038/s41467-022-29144-4 (PMC8938466; doi:10.1038/s41467-022-29144-4)
Supplement: Supplementary file 13 — Reporting Summary [file 41467_2022_29144_MOESM13_ESM.pdf]

nature research

Corresponding author(s): Yong Wang

Last updated by author(s): Feb 15, 2022

# Reporting Summary

Nature Research wishes to improve the reproducibility of the work that we publish. This form provides structure for consistency and transparency in reporting. For further information on Nature Research policies, see our Editorial Policies and the Editorial Policy Checklist.

## Statistics

For all statistical analyses, confirm that the following items are present in the figure legend, table legend, main text, or Methods section.

| n/a | Confirmed | |
|---|---|---|
| ☐ | ☒ | The exact sample size (*n*) for each experimental group/condition, given as a discrete number and unit of measurement |
| ☐ | ☒ | A statement on whether measurements were taken from distinct samples or whether the same sample was measured repeatedly |
| ☐ | ☒ | The statistical test(s) used AND whether they are one- or two-sided<br>*Only common tests should be described solely by name; describe more complex techniques in the Methods section.* |
| ☒ | ☐ | A description of all covariates tested |
| ☒ | ☐ | A description of any assumptions or corrections, such as tests of normality and adjustment for multiple comparisons |
| ☐ | ☒ | A full description of the statistical parameters including central tendency (e.g. means) or other basic estimates (e.g. regression coefficient) AND variation (e.g. standard deviation) or associated estimates of uncertainty (e.g. confidence intervals) |
| ☐ | ☒ | For null hypothesis testing, the test statistic (e.g. *F*, *t*, *r*) with confidence intervals, effect sizes, degrees of freedom and *P* value noted<br>*Give P values as exact values whenever suitable.* |
| ☒ | ☐ | For Bayesian analysis, information on the choice of priors and Markov chain Monte Carlo settings |
| ☒ | ☐ | For hierarchical and complex designs, identification of the appropriate level for tests and full reporting of outcomes |
| ☐ | ☒ | Estimates of effect sizes (e.g. Cohen's *d*, Pearson's *r*), indicating how they were calculated |

*Our web collection on statistics for biologists contains articles on many of the points above.*

## Software and code

Policy information about availability of computer code

| | |
|---|---|
| Data collection | Bowtie2 v2.4.1, SPAdes v.3.13, fastp v.0.2.0, SortMeRNA v.2.1, prefetch v.2.1.5, FastQC v0.11.8 |
| Data analysis | HMMER v.3.1b2, Qiime2 v.2019.7.0, Parallel-META v.3.5.1, MaxBin v.2.2.6, MetaBAT v.2.12.1, CONCOCT v.1.0.0, CheckM v.1.0.12, metaWRAP v.1.2.2, dRep v.1.4.3, GTDB-Tk v.1.0.2, SpecI v.1.0, MAFFT v.7.407, trimAl v.1.4, IQ-TREE v.1.6.11, RAxML v.8.2.12, iTOL v.4, bwa v.0.7.17, samtools v.1.9, CoverM v.0.4.0, R v.3.6, DIAMOND v.0.9.27.128, Prodigal v.2.6.3, KofamScan v.1.1.0, dbCAN v.2, eggNOG-mapper v.2.0.0, MEGAN v.6.18.5, GhostKOALA v.2.2, BLASTn v2.9.0, seqtk v1.3, in-house scripts used for data processing, analysis, statistics and data visualization are publicly available through GitHub(https://github.com/ucassee/Challenger-Deep-Microbes). |

For manuscripts utilizing custom algorithms or software that are central to the research but not yet described in published literature, software must be made available to editors and reviewers. We strongly encourage code deposition in a community repository (e.g. GitHub). See the Nature Research guidelines for submitting code & software for further information.

## Data

Policy information about availability of data

All manuscripts must include a data availability statement. This statement should provide the following information, where applicable:

- Accession codes, unique identifiers, or web links for publicly available datasets
- A list of figures that have associated raw data
- A description of any restrictions on data availability

The raw metagenome and metatranscriptome sequencing data, and MAG sequences generated in this study have been deposited in the NCBI database under the accession code PRJNA635214 [https://www.ncbi.nlm.nih.gov/bioproject/PRJNA635214]. The processed gene annotation and 16S miTag sequences are available at Figshare (https://doi.org/10.6084/m9.figshare.12979709). Accession numbers for the previous published metagenomes and MAGs used for comparisons can be found in Source data of Fig. 1b, and in the NCBI database under accession code PRJNA362212 [https://www.ncbi.nlm.nih.gov/bioproject/362212]. Public available

datasets used in this study include the checkM v.1.0.12 database (https://data.ace.uq.edu.au/public/CheckM_databases/), the GTDB database release 89 (https://data.gtdb.ecogenomic.org/releases/release89/), the SILVA 132 and 138 SSU database (https://www.arb-silva.de/download/archive/), HMM profiles of KEGG release 92.0 (https://www.genome.jp/ftp/db/kofam/archives/), MEROPS database release 12.1 (https://www.ebi.ac.uk/merops/download_list.shtml), dbCAN2 database (https://bcb.unl.edu/dbCAN2/download/Databases/), megan-map-Oct2019 database (https://software-ab.informatik.uni-tuebingen.de/download/megan6/old.html) and eggNOG 5.0 database (http://eggnog5.embl.de/download/eggnog_5.0/). Source data are provided with this paper. Correspondence and material requests should be directed to Y. Wang (wangy@idsse.ac.cn).

# Field-specific reporting

Please select the one below that is the best fit for your research. If you are not sure, read the appropriate sections before making your selection.

☒ Life sciences ☐ Behavioural & social sciences ☐ Ecological, evolutionary & environmental sciences

For a reference copy of the document with all sections, see nature.com/documents/nr-reporting-summary-flat.pdf

# Life sciences study design

All studies must disclose on these points even when the disclosure is negative.

| | |
|---|---|
| Sample size | Thirteen sediment cores were collected from both slope and bottom-axis sites in Challenger Deep at depths ranging from 5,400 m to 10,911 m. The north and south slope samples were collected at every ~1000m depth. Hence, our samples cover all depths of the both slopes of the Challenger Deep. We also obtained four replicates from different sites in the bottom-axis sites. From the sediment cores, we randomly select 2-4 layers from oxic and anoxic layers. Therefore, 37 subsamples were used for metagenome analysis. The 3 subsamples from one bottom-axis core were used for metatranscriptome analysis. |
| Data exclusions | No data were excluded from this manuscript. |
| Replication | Detection of arsenate, total arsenic, total selenium total mercury and porewater nutrient (NO3–, NO2–, and NH4+) concentrations were replicated independently by processing two replicate subsamples. All detections of two replications were performed within on month. All detections at replications were successful. According to the data and methods provided in the papers, all results can be reproduced. |
| Randomization | All sediment cores were used to represent both slope and bottom-axis sites in Challenger Deep and water depth profile. Subsamples of sediment cores by 2- or 3-centimeter layers were selected to represent sediment depth profile. We randomly selected 2-4 layers from oxic and anoxic layers from similar layer depths. For samples in each layer, they were homogenized for various treatments. |
| Blinding | Blinding was not applicable to this study. All methods and results that we used for analysis sediment samples are objective. |

# Reporting for specific materials, systems and methods

We require information from authors about some types of materials, experimental systems and methods used in many studies. Here, indicate whether each material, system or method listed is relevant to your study. If you are not sure if a list item applies to your research, read the appropriate section before selecting a response.

## Materials & experimental systems

| n/a | Involved in the study | |
|---|---|---|
| ☒ | ☐ | Antibodies |
| ☒ | ☐ | Eukaryotic cell lines |
| ☒ | ☐ | Palaeontology and archaeology |
| ☒ | ☐ | Animals and other organisms |
| ☒ | ☐ | Human research participants |
| ☒ | ☐ | Clinical data |
| ☒ | ☐ | Dual use research of concern |

## Methods

| n/a | Involved in the study | |
|---|---|---|
| ☒ | ☐ | ChIP-seq |
| ☒ | ☐ | Flow cytometry |
| ☒ | ☐ | MRI-based neuroimaging |
